# Effect of genetic mutations on outcomes of stem cell transplantation in children with hemophagocytic lymphohistiocytosis

**DOI:** 10.1038/s41409-025-02592-4

**Published:** 2025-04-22

**Authors:** Gülyüz Öztürk, Mehmet Akif Yeşilipek, Arzu Akçay, Vedat Uygun, Gülcihan Özek, Gülsün Karasu, Ebru Yılmaz, Fatma Demir Yenigürbüz, Seda Öztürkmen, Serap Aksoylar, İkbal Ok Bozkaya, Koray Yalçın, Başak Adaklı Aksoy, Ekrem Ünal, Burcu Akıncı, Hayriye Daloğlu, Barbaros Şahin Karagün, Savaş Kansoy, Namık Özbek, Elif İnce, Hacı Ahmet Demir, Müge Gündoğdu, Barış Malbora, Musa Karakükçü, Murat Elli, Arzu Akyay, Adalet Meral Güneş, Sinan Akbayram, Nazan Sarper, Buket Erer Del Castello, Volkan Hazar, Bülent Antmen

**Affiliations:** 1 Pediatric BMT Unit, Acıbadem Altunizade Hospital, Acıbadem University Faculty of Medicine, Istanbul, Turkey; 2 Pediatric BMT Unit, Medical Park Göztepe Hospital, Istanbul, Turkey; 3 Pediatric BMT Unit, Medical Park Antalya Hospital, Istinye University Faculty of Medicine, Antalya, Turkey; 4 Pediatric BMT Unit, Ege University Faculty of Medicine, Izmir, Turkey; 5 Pediatric BMT Unit, Erciyes University Faculty of Medicine, Kayseri, Turkey; 6 Pediatric BMT Unit, Ankara Bilkent City Hospital, University of Health Sciences, Ankara, Turkey; 7 Pediatric BMT Unit, Bahçelievler Medical Park Hospital, Altınbaş University Faculty of Medicine, İstanbul, Turkey; 8 Pediatric Hematology and Oncology Clinic, Medical Point Hospital, School of Health Sciences, Hasan Kalyoncu University, Gaziantep, Turkey; 9 Faculty of Health Sciences, Antalya Bilim Univercity, Antalya, Turkey; 10 Pediatric BMT Unit, Acibadem Adana Hospital, Adana, Turkey; 11 Pediatric BMT Unit, Ankara University Faculty of Medicine, Ankara, Turkey; 12 Pediatric BMT Unit, Memorial Ankara Hospital, Ankara, Turkey; 13 Pediatric BMT Unit, Memorial Bahçelievler Hospital, Istanbul, Turkey; 14 Pediatric BMT Unit, GOP Hospital, Yüzüncü Yıl University Faculty of Medicine, Istanbul, Turkey; 15 Pediatric BMT Unit, İstanbul Medipol University Faculty of Medicine, Istanbul, Turkey; 16 Pediatric BMT Unit, İnönü University Faculty of Medicine, Malatya, Turkey; 17 Pediatric BMT Unit, Uludağ University Faculty of Medicine, Bursa, Turkey; 18 Pediatric BMT Unit, Gaziantep University Faculty of Medicine, Gaziantep, Turkey; 19 Pediatric BMT Unit, Kocaeli University Faculty of Medicine, Kocaeli, Turkey; 20 Pediatric BMT Unit, Yeditepe University Faculty of Medicine, Istanbul, Turkey; 21 Pediatric Pediatric Hematology and Oncology Clinic, Medstar Yıldız Hospital, Antalya, Turkey

**Keywords:** Immunological deficiency syndromes, Haematopoietic stem cells

## Abstract

Primary hemophagocytic lymphohistiocytosis (p-HLH) can be cured with allogeneic haematopoietic stem cell transplantation (allo-HSCT). It remains unclear whether HSCT outcomes are affected by the presence of different genetic mutations. We used data obtained from children who underwent allo-HSCT for HLH to examine the effects of genetic mutations on HSCT outcomes. Data from 153 paediatric patients in 18 paediatric stem cell centres were retrospectively evaluated. Patients were divided into four groups: 1) with PRF1 mutation (*n* = 46), 2) with UNC13D mutation (*n* = 38), 3) with STX11/STXBP2 mutation (*n* = 25) and 4) with Griscelli syndrome type 2/ Chediak–Higashi syndrome (GS2/CHS) diagnosis (*n* = 44). Statistical analysis showed no difference between the subgroups in terms of engraftment, VOD, acute GVHD, chronic GVHD, TRM, OS and EFS rates. The most important factor affecting OS and EFS in all genetic subgroups was remission status before HSCT. The 5-year EFS values for children with mutations in PRF1, UNC13D, STX11/STXBP2 and GS2/CHS were 71%, 66.6%, 74% and 66.7, respectively (log-rank >0.05). However, with prospective studies covering more patients, and creating different genetic subgroups by performing more detailed genetic analyses, special approaches for different genetic subgroups can be revealed in the future.

## Introduction

Hemophagocytic lymphohistiocytosis (HLH) is an aggressive and life-threatening hyperinflammation syndrome characterised by dysregulated activation and proliferation of T cells, natural killer cells and macrophages [1–4]. HLH has been traditionally categorised as primary or genetic and secondary. Primary-HLH (p-HLH) are a heterogeneous group, the most common form of which is caused by genetic defects in familial HLH (f-HLH) genes (perforin [PRF1] [5], Unch [UNC13D] [6, 7] and syntaxin [STX11 and STXBP2]) [8]. Abnormal cytotoxic lymphocyte cytotoxicity is responsible for pathogenesis [9]. In the other form, loss-of-function mutations in the RAB27A, LYST, and AP3B1 genes, cause problems in the structure of the cytotoxic granules or transport of the granules through the cytoplasm. These diseases are also cause genetic HLH and are known as Griscelli syndrome type 2 (GS2) [10], Chediak–Higashi syndrome (CHS) [11] and Hermansky–Pudlak syndrome type 2 [12], respectively. However, other primary immunodeficiencies have a high risk of HLH [13–15], and some metabolic diseases may be complicated by HLH [16, 17]. Due to the wide genetic heterogeneity, it is not surprising that the time of onset, clinical presentation and severity of HLH differ in patients with genetic mutations.

In p-HLH, cure can be achieved with allogeneic haematopoietic stem cell transplantation (allo-HSCT) following treatment protocols containing high immuno- and myelosuppressive drugs, such as HLH-94, and later HLH-2004 protocols [18]. Allo-HSCT is also performed after salvage treatment in relapsed or refractory secondary HLH [19, 20]. According to the results of the HLH-94 protocol (*n* = 65), the 3-year probability of overall survival (OS) post-HSCT was 62% (±12%), but transplant-related mortality (TRM) was found to be higher than other nonmalignant diseases [21]. In the HLH-2004 study (*n* = 187), the largest prospective study on HSCT in HLH, 5-year OS and event-free survival (EFS) after HSCT were 66% and 60% in the entire group and 71% and 62% in the verified f-HLH group, respectively. However, 54% of the patients died due to TRM [22]. Consistently, recent allo-HSCT approaches have focused on reducing HSCT toxicity and increasing survival [23–26].

Factors such as remission status before HSCT, time between diagnosis and HSCT, and conditioning regimen (myeloablative conditioning regimen [MAC] / reduced-intensity conditioning [RIC] / reduced-toxicity conditioning [RTC]) used in HSCT affect the success of HSCT [23–26]. However, it is not clear whether HSCT is affected by genetic mutations.

We aimed to examine the effects of genetic mutations on HSCT outcomes in children who underwent allo-HSCT for p-HLH.

## Patients and methods

### Patients

The study was designed by the Turkish Paediatric Bone Marrow Transplantation Study Group. Study approval was obtained from the Ethics Committee of Acibadem Mehmet Ali Aydinlar University Faculty of Medicine (approval no: 2021-19/06). All methods were performed in accordance with the relevant guidelines and regulations. The informed consent form was obtained from parents for allo-HSCT. Transplantations were performed between March 2008 and October 2020. Inclusion criteria were as follows: (a) allo-HSCT performed due to p-HLH, whose mutation was determined by genetic analysis, (b) aged <18 years at allo-HSCT and (c) first allo-HSCT. Exclusion criteria were as follows: patients who did not fulfil all inclusion criteria.

Data from 191 paediatric patients with HLH who underwent allo-HSCT in 18 paediatric stem cell centres in Turkey were retrospectively evaluated. p-HLH could not be verified in 17 patients (15 and 2 patients were screened for four and three disease-causing genes, respectively). Genetic analysis could not be performed in 21 patients. A total of 38 patients were excluded from the study. The diagnosis of p-HLH in 153 patients was confirmed by genetic analysis. The patients were divided into four groups: 1) with the PRF1 mutation (*n* = 46), 2) with the UNC13D mutation (*n* = 38), 3) with the STX11/STXBP2 mutation (*n* = 25), 4) with GS2/CHS diagnosis (*n* = 44). Patient characteristics were detailed in Table 1.

**Table 1:** Patient characteristics for all children, children with verified f-HLH (PRF1, UNC13D, STX11/STXBP2) and children with GS2/CHS.

Characteristics	All patients (*n* = 153)	Genetic type				*P* value
		PRF1 (*n* = 46)	UNC13D (*n* = 38)	STX11/STXBP2 (*n* = 25)	GS2/CHS (*n* = 44)	
Female/Male	73/80	20/26	19/19	13/12	21/23	0.89
Age at diagnosis (mo) Median (min, max)	6 (0.1–204)	3.5 (0.1–140)	3.5 (1–88)	16 (1–172)	9.5 (0.5–204)	* 0.002
Age at diagnosis (*n*, %) <1 year old ≥1 years old	106 (69%) 47 (31%)	36 (78) 10 (22)	32 (84) 6 (16)	12 (48) 13 (52)	26 (59) 18 (41)	* 0.004
Age at HSCT (mo) Median (min, max)	18 (1–215)	10 (1–165)	13.5 (5–54)	41 (9–177.5)	32 (1–215)	* <0.001
Age at HSCT (*n*, %) <1 year old 1–5 years old ≥5 years old	59 (39%) 60 (39%) 34 (22%)	30 (65) 7 (15) 9 (20)	15 (39.5) 22 (58) 1 (2.5)	3 (12) 10 (49) 12 (48)	11 (25) 21 (48) 12 (27)	** <0.001
Time to HSCT from HLH diagnosis (mo) Median (min, max)	8.1 (0.1–94)	5.1 (0.1–94)	7.8 (1.3–45.4)	20.3 (3.4–91.2)	13.4 (0.5–94)	*** <0.001
Time to HSCT from HLH diagnosis (*n*, %) <6 months ≥6 months	57 (37%) 96 (63%)	27 (59) 19 (41)	12 (31.5) 26 (68.5)	3 (12) 22 (88)	15 (34) 29 (66)	** 0.001
Remission status at HSCT^a^						
CR (*n*, %)	142 (93%)	31 (63.5)	24 (63)	13 (52)	28 (63.5)	
AD	11 (7%)	3 (6.5)	3 (8)	3 (12)	2 (4.5)	0.71
Treatment before HSCT (*n*)						
HLH 2004/The other/No treatment	133/14/6	42/4/-	36/1/1	22/1/2	34/8/2	0.07

*HLH* Hemophagocytic lymphohistiocytosis, *f-HLH* familial HLH, *PRF1* perforin1, *STX11* syntaxin 11, *STXBP2* syntaxin binding protein 2, *GS2* Gricelli syndrome type 2, *CHS* Chediak–Higashi syndrome, *HSCT* Hematopoietic stem cell transplantation, *CR* complete remission, *AD* Active disease.

*In children with the PRF1 and UNC13D mutations, the age at diagnosis of HLH, the age of HSCT were significantly lower and the rate of diagnosis before the age of 1 was higher than those in other groups (*p* < 0.05).

**The rate of HSCT performed within 6 months after diagnosis and before 1 year of age in patients with perforin mutation was significantly higher than in all other groups (*p* < 0.05).

***The time from diagnosis to HSCT was significantly shorter in the group with the PRF1 mutation than in other groups and was shorter in the UNC13D group than in the STX11/STXBP2 group (*p* < 0.001).

^a^Absence of fever, splenomegaly, cytopenia (cutoff for platelets: 100.000/mm^3^ and cutoff for absolute neutrophil count: 1000/mm^3^), hyperferritinemia (cutoff: 2000 ng/mL), hypofibrinogenemia (cut off: 150 mg/mL) and hemophagocytosis on bone marrow (BM) smear was defined as complete remission (CR). One missing parameter was allowed. Active disease (AD) was defined as having >3 abnormal results. The patients between the two groups were considered to be in partial remission. In patients with central nervous system involvement at initial diagnosis, CR was accepted as the absence of active lesions on cranial MRI and the absence of cell and protein increase in the cerebral spinal fluid [22].

### HLH disease status before HSCT

The activity of HLH was evaluated with both clinical and laboratory parameters before starting the conditioning regimen. Absence of fever, splenomegaly, cytopenia, hyperferritinemia [27–29], hypofibrinogenemia and hemophagocytosis on bone marrow (BM) smear was defined as complete remission (CR). One missing parameter was allowed. Active disease (AD) was defined as having >3 abnormal results [22]. All of patients, 142 (93%) were in CR (CR1 = 96, CR2 = 35 and ≥CR3 = 11) before HSCT and 133 were treated with the HLH-2004 protocol (Table 1).

### Donor typing

In total, 73 (48%) patients had matched related donors (MRDs), 49 (32%) (*n* = 20; 10/10 matched and *n* = 29; 9/10 matched) had matched unrelated donors, 18 (12%) had haploidentical donors, and 13 (8%) (*n* = 7; 6/6 matched, *n* = 5; 5/6 matched and *n* = 1; 4/6 matched) had unrelated umbilical cord blood donors (Table 2).

**Table 2:** Details on HSCT procedure for all children, children with verified f-HLH (PRF1, UNC13D, STX11/STXBP2) and children with GS2/CHS.

Characteristics (*n*, %)	All patients (*n* = 153)	Genetic type				*P* value
		PRF1 (*n* = 46)	UNC13D (*n* = 38)	STX11/STXBP2 (*n* = 25)	GS2/CHS (*n* = 44)	
Type of donor (*n*, %)						0.93
MRD (MSD/MFD)	73 (46/27) (48%)	21 (11/10) (46)	17 (10/7) (45)	13 (9/4) (52)	22 (17/5) (50)	
MUD (10/10, 9/10)	49 (20/29) (32%)	12 (4/8) (26)	14 (3/11) (36.5)	7 (3/4) (28)	14 (6/8) (32)	
MUD - UCB	13 (8%)	6 (13)	4 (10.5)	2 (8)	3 (7)	
Haploidentical donor	18 (12%)	7 (15)	3 (8)	3 (12)	5 (11)	
Conditioning regimen						*
MAC/RIC (*n*,%)	141/12 (92%/8%)	46/- (100/-)	33/5 (87/13)	24/1 (96/4)	38/6 (86/14)	0.047
Busulfan-based	126 (82%)	36 (78)	30 (79)	21 (84)	39 (89)	
Flu/Mel; Flu/Mel/TT	27 (22;5)(18%)	10 (8;2)(22%)	8 (6;2)(21%)	4 (3;1)(16%)	5 (4;1) (11%)	
GVHD prophylaxis (*n*,%)						0.33
CSA alone	20 (13%)	6 (13)	8 (21)	4 (16)	2 (4.5)	
CSA + MTX	99 (65%)	25 (54)	25 (66)	16 (64)	33 (75)	
CSA + MMF	6 (4%)	3 (7)	1 (2.5)	–	2 (4.5)	
CSA + CS	14 (9%)	6 (13)	3 (8)	1 (4)	4 (9)	
The other	14 (9%)	6 (13)	1 (2.5)	4 (16)	3 (7)	
ATLG (*n*,%)						0.21
Yes	109 (71%)	28 (61)	31 (81.5)	18 (72)	32 (73)	
No	44 (29%)	18 (39)	7 (18.5)	7 (28)	12 (27)	
Graft type (*n*,%)						0.57
Bone marrow	89 (58%)	30 (65)	21 (55.5)	13 (52)	25 (57)	
PBSC	45 (29%)	9 (20)	13 (34)	8 (32)	15 (34)	
CBC	15 (10%)	6 (13)	4 (10.5)	2 (8)	3 (7)	
Bone marrow + PBCS	4 (3%)	1 (2)	21 (55.5)	2 (8)	1 (2)	
CD 34+ cells in BM/PBSC (×10^6^/BW), median (range)	6.6 (0.8–42.3)	6.51 (0.8–42.3)	6.5 (2.24–28)	5.82 (1.8–24.3)	7.25 (0.86–35.7)	0.87
TNCs in BM (×10^8^/BW), median (range)	7 (1.4–118.8)	7 (2.1–118.7)	6.4 (0.8–31.1)	6 (2–21.5)	7.5 (1.15–78.85)	0.44
CD 34+ cells in CBC (×10^5^/BW), median (range)	9 (1.1–29)	11.3 (4.9–29)	3.8 (1.1–26)	6.12 (3.7–8.5)	5.6 (3–8.6)	0.22
TNCs in CBC (×10^7^/BW, median (range)	16.5 (2.47–26)	18.85 (10.5–26)	2.47 (2.2–8.5)	2.58 (1.9–7.5)	8.95 (4.1–13)	0.09

*HLH* Hemophagocytic lymphohistiocytosis, *f-HLH* familial HLH, *PRF1* perforin1, *STX11* syntaxin 11, *STXBP2* syntaxin binding protein 2, *GS2* Gricelli syndrome type 2, *CHS* Chediak–Higashi syndrome, *MRD* matched related donor, *MFD* matched family donor, *MSD* matched sibling donor, *MUD* matched unrelated donor, *UCB* umbilical cord blood, *MAC* myeloablative conditioning, *RIC* reduced intensity conditioning, *Flu* fludarabine, *Mel* melphalan, *TT* thiotepa, *GVHD* graft-versus-host disease, *CSA* cyclosporine, *MTX* methotrexate, *MMF* mycophenolate mofetil, *CS* corticosteroid, *ATLG* anti-T lymphocyte globulin, *PBSC* peripheral blood stem cell, *CBC* cord blood cell, *BM* bone marrow, *TNC* total nucleated cell.

*MAC conditioning regimen was used in all of the perforin group, but RIC was not used (*p* < 0.05).

### Conditioning regimen, GVHD prophylaxis, stem cell source

Transplantation procedures were planned by the transplantation team in line with the recommendations of the HLH-2004 treatment protocol [22] or EBMT/ESID guidelines for HSCT for primary immunodeficiencies [30] or the experience of the centres. A dose of 16 mg/kg busulfan was considered MAC, and lower doses were considered RIC. Pharmacokinetic studies could be performed for busulfan in some of the centres. In total, 141 (92%) patients received a MAC regimen (busulfan based = 121) and 12 (8%) received a RIC regimen (busulfan based = 5) (Table 2).

A total of 139 patients received cyclosporine for GVHD prophylaxis as monotherapy (*n* = 20) or combination (*n* = 119). Anti-human T lymphocyte immunoglobulin (Grafalon) was used in 71% (*n* = 109) of the patients (Table 2).

Stem cell source was BM in 89 (58%) patients. Details of the source and numbers of infused stem cells are in Table 2. Alpha beta depleted grafts were used in 10/18 patients who underwent haploidentical allo-HSCT.

### Definitions and assessment of engraftment and chimerism

Engrafment, aGVHD and cGVHD, VOD and primary and secondary graft failure were defined according to the EBMT Guideline [31–34]. Haematopoietic chimerism was assessed using peripheral blood samples of the patient and donor through short-tandem repeated sequence PCR DNA fingerprinting for all pairs.

### Statistical analysis

The Statistical Package for Social Sciences statistical package program (SPSS version 16.0) was used for data analysis. The incidence of cGVHD was calculated after excluding all patients with a follow-up period of <100 days. The effects of multiple variables were analysed by logistic regression analysis. The incidence of TRM and relapse was estimated using the cumulative incidence function. OS rate and EFS were evaluated with Kaplan–Meier survival analysis. Factors affecting survival were analysed by Cox regression analysis. Regression analysis comparisons were made according to perforin mutation group. *P* values of <0.05 were considered significant.

## Results

### Patients characteristics

Of the 153 patients with HLH enroled in the study, 80 were males and 73 were females with a median age of 18 months (range: 1–215 months) at HSCT. In children with the PRF1 and UNC13D mutations, the age at diagnosis of HLH, the age of HSCT were significantly lower and the rate of diagnosis before the age of 1 was higher than those in other groups (*p* < 0.05). The time from diagnosis to HSCT was significantly shorter in the group with the PRF1 mutation than in other groups (median: 5.1 months). The rate of HSCT performed within 6 months after diagnosis and before 1 year of age in patients with perforin mutation was significantly higher than in all other groups (*p* < 0.05).

There was no statistical difference between the groups in terms of donor type, treatment used before HSCT, remission status of patients at HSCT and GVHD prophylaxis used, graft source and number of CD34+ cells. However, in terms of conditioning regimen, the perforin group was different from the other groups as MAC conditioning regimen was used in all of patients (*p* < 0.05). Details in Tables 1 and 2.

### Engraftment and complications (VOD, GVHD, transplant-associated thrombotic microangiopathy [TA-TMA] and noninfectious pulmonary complications)

Neutrophil engraftment and platelet engraftment were achieved in 95% and 90% of the patients, respectively. No difference was observed between the groups in terms of engraftment rates and engraftment days (Table 3).

**Table 3:** Comparison of transplantation outcomes, for all children, children with verified f-HLH (PRF1, UNC13D, STX11/STXBP2) and children with GS2/CHS.

	All patients (*n* = 153)	Genetic type				*P* value
		PRF1 (*n* = 46)	UNC13D (*n* = 38)	STX11/STXBP2 (*n* = 25)	GS2/CHS (*n* = 44)	
Neutrophil engrafment						
Yes (*n*, %)	145 (95%)	44 (96)	37 (97)	22 (88)	42 (95.5)	0.4
Platelet engrafment						
Yes (*n*, %)	138 (90%)	40 (87)	37 (97)	23 (92)	38 (86)	0.31
Engraftment, median						
Neutrophil (range, days)	15 (8–48)	14 (8–35)	15 (9–48)	16 (10–25)	15 (10–31)	0.2
Platelet (range, days)	20 (8–126)	20 (8–126)	23 (10–72)	21 (10–60)	20 (9–45)	0.85
Defibrotide prophylaxis VOD	85 (56%)	28 (61)	25 (67)	15 (60)	17 (39)	0.59
Yes (*n*, %)	33 (21.6%)	10 (22)	8 (21)	5 (20)	10 (23)	0.9
Defibrotide prophylaxis in those who develop VOD	21 (64%)	6 (60)	5 (63)	5 (100)	5 (50)	0,73
Acute GVHD (*n*,%)						
Yes	53 (35%)	17 (35.5)	15 (40)	9 (33)	15 (32.5)	0.57
Grade I-II	24 (16%)	5 (11)	9 (24)	5 (21)	5 (11.5)	
Grade III-IV	29 (19%)	11 (24.5)	6 (16)	3 (12)	9 (21)	
Chronic GVHD (*n*,%)						
No	104 (81%)	33 (82.5)	26 (74)	18 (82)	32 (89)	0.34
Yes (Limited/extended)	24 (15/9) (19%)	7 (5/2) (17.5)	9 (6/3) (26)	4 (3/1) (18)	4 (1/3) (11)	
Donor chimerism at day 30 (*n*, %) (complete/mix)	136 (89%) (102/34)	39 (85) (30/9)	33 (87) (24/9)	24 (96) (16/8)	40 (91) (32/8)	0.42
Donor chimerism at day 180, (*n*,%) (complete/mix)	119 (78%) (88/31)	35 (76) (26/9)	30 (79) (24/6)	22 (88) (14/8)	32 (73) (24/8)	0.32
Graft failure (*n*,%) (primary/secondary)	13 (9%) (8/5)	4 (3/1)	5 (4/1)	1 (0/1)	3 (1/2)	0.76

*HLH* Hemophagocytic lymphohistiocytosis, *f-HLH* familial HLH, *PRF1* perforin1, *STX11* syntaxin 11, *STXBP2* syntaxin binding protein 2, *GS2* Gricelli syndrome type 2, *CHS* Chediak–Higashi syndrome, *p-HLH* primary HLH, *VOD* veno-occlusive disease, *GVHD* graft-versus-host disease.

Defibrotide prophylaxis was administered in 56% (*n* = 85) of the patients, and VOD developed in 21.6% (*n* = 33) of the patients. There was no statistically significant difference between the groups in terms of defibrotide prophylaxis use and VOD development (*p* = 0.59 and *p* = 0.9, respectively). The rate of VOD was not different between those who did and did not receive defibrotide prophylaxis in all patients and in groups with different genetic mutations (*p* = 0.73) (Table 3). In addition, no statistically significant difference was observed in the severity of VOD in the whole group and in the subgroups between patients who used and did not use prophylactic defibrotide.

Of the 153 patients, 53 (35%) experienced aGVHD, including 24 (16%) with grade I/II and 29 (19%) with grade III/IV aGVHD. The frequency of cGVHD was 19% (24/128 patients). The incidences of aGVHD and cGVHD were statistically not different between the groups (*p* = 0.57 and *p* = 0.34) (Table 3). In multivariate analysis, no effect of genetic mutation on myeloid engraftment, platelet engraftment, VOD, aGVHD and cGVHD development was detected (*p* > 0.05) Table 4A). The analysis results regarding the variables affecting these conditions in all patients are also shown in Table 4A.

**Table 4:** A: The effect of subgroups of HLH with genetic mutations on engraftment, VOD, acute GVHD, chronic GVHD, survival and 100-day TRM by multivariate analysis, and variables that have a statistically significant effect on these results. B: Hazard ratios, 95% CIs and *p* values from Cox proportional hazards models for subgroups of HLH with genetic mutations.

A.	Genetic type			
	UNC13D (*n* = 38) Odds ratio (95% CI; *p*)	STX11/STXBP2 (*n* = 25) Odds ratio (95% CI; *p*)	GS2/CHS (*n* = 44) Odds ratio (95% CI; *p*)	Variables with statistically significant effect Odds ratio (95% CI; *p*)^b^
Neutrophil engrafment^a^	1.5 (*P* = 0.98)	1.1 (*P* = 0.51)	0.0 (*P* = 0.38)	- Remission status at HSCT: (AD versus CR) CR: 62 (10–1000; 0.001)
Platelet engrafment	1.7 (0.2–17.7; 0.63)	62 (0.8–5170; 0.06)	18 (0.65–492; 0.08)	
VOD	0.53 (0.12–2.3; 0.4)	0.4 (0.1–1.8; 0.26)	0.57 (0.1–2.9; 0.51)	- Type of donor: (MFD versus haplo) Haploidentical donor: 6 (1.4–27; 0.017)
Acute GVHD	2.2 (0.62–7.8; 0.21)	1.8 (0.51–6.2; 0.35)	1.8 (0.45–7; 0.41)	-
Chronic GVHD	2.1 (0.24–19; 0.5)	7.5 (0.9–64; 0.06)	2.5 (0.25–26.6; 0.43)	- Acute GVHD: (No versus yes) Yes: 7.5 (2.4–24; 0.001)
Relapse	0.3 (0.01–5; 0.4)	3 (0.3–28; 0.35)	0.15 (0.01–4; 0.26)	- Remission status at HSCT: (CR versus AD) AD: 189 (5–7072; 0.005) - Time to HSCT from diagnosis: (<6 versus ≥6 mo) ≥6 months: 34 (1.7–682; 0.021) - ATLG: (No versus yes) Yes: 35.5 (1.7–719; 0.02) - GVHD prophylaxis (CSA alone versus CSA + MTX) CSA alone: 50 (3.3–1000; 0.01)
TRM at day 100; (*n*,%)	0.36 (0.07–1.8;0.22)	0.21 (0.03–1.7; 0.15)	0.21 (0.03–1.6; 0.13)	- Remission status at HSCT: (AD versus CR) AD: 15.5 (2.2–109; 0.006)
Patients survival (alive vs. dead)	26.7 (1–657; 0.04)	3.7 (0.49–28; 0.2)	0.9 (0.13–6.6; 0.9)	- Remission status at HSCT: (AD versus CR) AD: 14 (2–97; 0.008) - Graft failure: (Yes versus no) Yes: 10 (2.5–40; 0.001)
**B.**				
	HR (95% CI; *p*)	HR (95% CI; *p*)	HR (95% CI; *p*)	
OS^c^	0.8 (0.24–2.7; 0.74)	1.8 (0.5–7; 0.36)	1.4 (0.5–4; 0.5)	- Remission status at HSCT: (CR versus AD) AD: 9.6 (4–23.6; 0.0001)
EFS^c^	1.3 (0.4–4; 0.6)	1.7 (0.4–6.7; 0.4)	1.7 (0.6–5; 0.3)	- Remission status at HSCT: (CR versus AD) AD: 10.3 (4–26; 0.0001)

Comparison was made according to perforin mutation group.

*HLH* Hemophagocytic lymphohistiocytosis, *p-HLH* primary HLH, *STX11* syntaxin 11, *STXBP2* syntaxin binding protein 2, *GS2* Gricelli syndrome type 2, *CHS* Chediak–Higashi syndrome, *TRM* transplant-related mortality, *VOD* veno-occlusive disease, *CR* complete remission, *AD* Active disease, *MFD* matched family donor, *GVHD* graft-versus-host disease, *CSA* cyclosporine, *MTX* methotrexate, *ATLG* anti-T lymphocyte globulin, *OS* overall survival, *EFS* event free survival, *HR* hazard ratio, *CI* confidence interval.

^a^With the available data, the spss program could not give the 95% CI values, so only *p* value was given.

^b^Multivariate analysis was performed on all patients and added as supplementary data.

^c^OS was calculated from the time of HSCT to the last follow-up time or death or second HSCT. Death, relapse or graft failure were defined as events.

In all patients, TA-TMA occurred in 2% (*n* = 3) and noninfectious pulmonary complications in 4.6% (*n* = 7), and no statistically significant difference was found between the subgroups in terms of these rates.

### Chimerism

Complete chimerism at day 30 was reported in 102 (71%) children, and mixed chimerism was reported in 34 (24%) (nondonor chimerism = 8 (5%) and no data/early death = 9). Graft failure developed in 13 (9%) (primary = 8 and secondary = 5) of 144 patients, and secondary poor graft function developed in two patients (patients who died in the first 30 days were excluded). The rates of graft failure and rates of donor chimerism at day 30 and at day 180 were statistically not different between the groups (*p* = 0.76, *p* = 0.42 and *p* = 0.32) (Table 3). Donor lymphocyte infusion was performed in seven patients with mixed chimerism, and complete chimerism was achieved in four patients. CD34+ cell infusion was performed in four patients (mixed chimerism = 2, secondary graft failure = 1 and secondary poor graft function = 1). Three children had a second HSCT.

### Survival

**Fig. 1:**
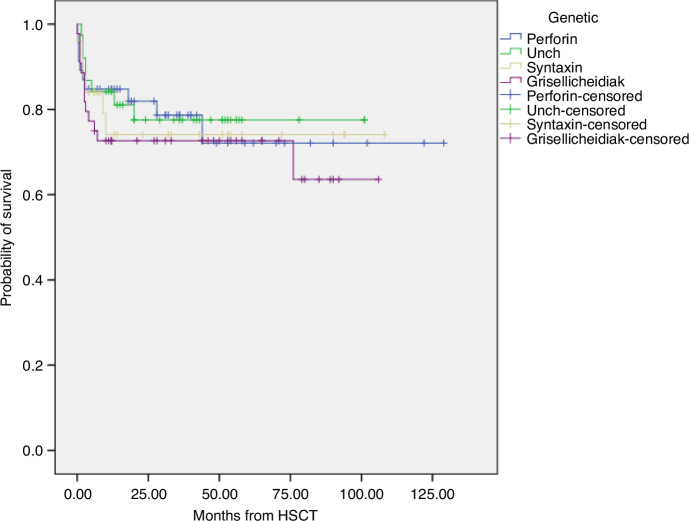
Kaplan–Meier estimates of overall survival (OS) after HSCT in different genetic subgroups. OS was calculated from the time of HSCT to the last follow-up time or death or second HSCT. For children with mutations in PRF1, UNC13D, STX11/STXBP2 and GS2/CHS, 5-year OS post-HSCT values were 72% (95% CI, 63–81), 77.6% (95% CI, 70–85), 74% (95% CI, 65–83) and 72.7% (95% CI, 66–79), respectively (log-rank >0.05). PRF1 perforin1, STX11 syntaxin 11, STXBP2 syntaxin binding protein 2, GS2 Gricelli syndrome type 2, CHS Chediak–Higashi syndrome, HSCT Hematopoietic stem cell transplantation.

**Fig. 2:**
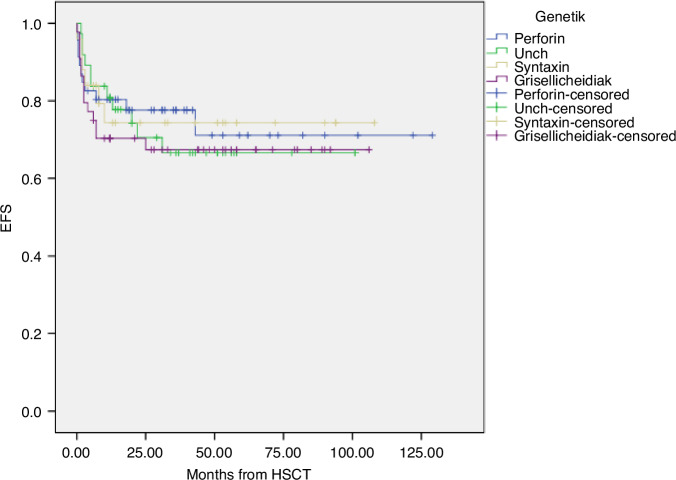
Kaplan–Meier estimates of event free survival after HSCT in different genetic subgroups. Death, relapse or graft failure were defined as events. Five-year EFS values for children with mutations in PRF1, UNC13D, STX11/STXBP2 and GS2/CHS were 71% (95% CI, 63–80), 66.6% (95% CI, 58–75), 74% (95% CI, 65–83) and 66.7% (95% CI, 60–75), respectively (log-rank >0.05). EFS Event free survival, PRF1 perforin1, STX11 syntaxin 11, STXBP2 syntaxin binding protein 2, GS2 Gricelli syndrome type 2, CHS Chediak–Higashi syndrome, HSCT Hematopoietic stem cell transplantation.

Patients were followed-up for a median of 28 months (range: 1 day to 129 months), with a follow-up of ≥3 years in 63. A total of 116 (76%) children survived. For children with mutations in PRF1, UNC13D, STX11/STXBP2 and GS2/CHS, 5-year OS post-HSCT values were 72% (95% CI, 63–81), 77.6% (95% CI, 70–85), 74% (95% CI, 65–83) and 72.7% (95% CI, 66–79), respectively (log-rank >0.05) (Fig. 1). In total, 16 (10.5%) patients developed relapse at a median of 7 months (range: 1.5–43 months) after HSCT. Five-year EFS values for these genetic subgroups were 71% (95% CI, 63–80), 66.6% (95% CI, 58–75), 74% (95% CI, 65–83) and 66.7% (95% CI, 60–75), respectively (log-rank >0.05) (Fig. 2). There was no statistical difference between the groups in terms of OS and EFS at day 100, 1 year and 5 year (Table 5). Cox regression analysis revealed that the genetic mutations of the patients did not affect OS and EFS (*p* > 0.05) (Table 4B). In all patients and genetic subgroups, OS and EFS were statistically significantly lower in patients who underwent HSCT in the presence of AD than in those who underwent HSCT in remission. OS and EFS were higher in the STX11/STXBP2 group in those who underwent HSCT at age ≥5 years compared to younger ages. Details of some variables affecting OS and EFS are summarized in a supplementary data (Table 6).

**Table 5:** Comparison of relapse, TRM, survival and EFS for all children, children with verified f-HLH (PRF1, UNC13D, STX11/STXBP2) and children with GS2/CHS.

	All patients (*n* = 153)	Genetic type				*P* value
		PRF1 (*n* = 46)	UNC13D (*n* = 38)	STX11/STXBP2 (*n* = 25)	GS2/CHS (*n* = 44)	
Follow-up median (range, months)	28 (0.03–129)	29.5 (0.13–129)	26.5 (1.5–101)	23 (0.06–108)	29.5 (0.01–106)	0.96
Last status (*n*, %)						
Alive	116 (76%)	36 (78)	30 (79)	19 (76)	30 (70)	0.79
Dead	37 (24%)	10 (22)	8 (21)	6 (24)	14 (30)	
Relapse (*n*, %)	16 (10,5%)	4 (9)	7 (18)	2 (8)	3 (7)	0.32
TRM at day 100; (*n*, %)	22 (59,5%)	7 (100)	4 (90)	3 (75)	8 (89)	0.8
TRM at the end of follow-up; (*n*, %)	29 (78%)	8 (80)	5 (62.5)	4 (66.5)	12 (86)	0.36
OS;% (95% CI) (no.events)						
At day 100	84 (80–86) (25)	85 (79–90) (7)	87 (81–92) (5)	84 (77–91) (4)	79.5 (73–86) (9)	0.82
At 1 year	80 (77–83) (31)	85 (79–90) (7)	84 (78–90) (6)	74 (65–83) (6)	72.7 (66–79) (12)	
At 5 year	79 (75–83) (32)	72 (63–81) (10)	77.6 (70–85) (8)	74 (65–83) (6)	72.7 (66–79) (13)	
Estimated mean survival time (months) (95% CI)	96 (86–105)	97.5 (80–115)	80 (67–93)	81 (63–100)	75 (61–89)	
EFS;% (95% CI) (no. events)						
At day 100	84 (81–87) (25)	82.6 (77–88) (8)	89 (84–94) (4)	84 (77–91) (4)	79 (73–86) (9)	0.87
At 1 year	77 (74–80) (35)	80.4(74–86) (9)	81 (74–87) (7)	74 (65–83) (6)	69.6 (63–77) (11)	
At 5 year	70 (66–74) (42)	71 (63–80) (11)	66.6 (58–75) 11)	74 (65–83) (6)	66.7 (60–75) (14)	

*HLH* Hemophagocytic lymphohistiocytosis, *f-HLH* familial HLH, *PRF1* perforin1, *STX11* syntaxin 11, *STXBP2* syntaxin binding protein 2, *GS2* Gricelli syndrome type 2, *CHS* Chediak–Higashi syndrome, *p-HLH* primary HLH, *TRM* transplant-related mortality, *OS* overall survival, *EFS* event free survival.

**Table 6:** Kaplan–Meier estimates of OS and EFS for all children, children with verified f-HLH (PRF1, UNC13D, STX11/STXBP2) and children with GS2/CHS.

Variable	All patients (*n* = 153) 5-y pSu (95% CI) (no. evaluated/no.events)		Genetic type							
			PRF1 (*n* = 46) 5-y pSu (95% CI) (no. evaluated/no.events)		UNC13D (*n* = 38) 5-y pSu (95% CI) (no. evaluated/no.events)		STX11/STXBP2 (*n* = 25) 5-y pSu (95% CI) (no. evaluated/no.events)		GS2/CHS (*n* = 44) 5-y pSu (95% CI) (no. evaluated/no.events)	
	OS	EFS	OS	EFS	OS	EFS	OS	EFS	OS	EFS
Remission status at HSCT	*P* < 0.0001	*P* < 0.0001	*P* < 0.0001	*P* < 0.0001	*P* < 0.0001	*P* < 0.0001	*P* = 0.6	*P* = 0.6	*P* = 0.002	*P* < 0.0001
CR	79(75–83)(142/27)	73(69–77)(142/34)	77 (68–86)(43/7)	76(68–84)(43/8)	84(78–91) (35/5)	70(62–79)(35/9)	75 (65–85) (22/5)	75(65–85)(22/5)	76 (69–83) (42/11)	70 (63–78) (42/12)
Active disease	18 (7–30)(11/9)	20 (8–32)(10/8)	(3/3)^a^	(3/3)^a^	(3/3)^a^	(2/2)^a^	(3/1)^a,b^	(3/1)^a,b^	(2/2)^a^	(2/2)^a^
Time to HSCT from diagnosis	*P* = 0.86	*P* = 0.42	*P* = 0.52	*P* = 0.35	*P* = 0.6	*P* = 0.28	*P* = 0.3	*P* = 0.3	*P* = 0.17	*P* = 0.3
<6 months	75 (69–81)(57/13)	76 (70–81)(57/13)	79 (71–88) (27/5)	81(74–88)(27/5)	79 (75–82) (12/2)	79(55–92)(12/2)	(3/–)	(3/–)	59 (56–62) (15/6)	59 (46–72) (15/6)
≥6 months	74 (69–79) (96/24)	66 (61–71) (95/29)	61 (59–63) (19/5)	57(42–72)(19/6)	76 (72–85) (26/6)	61(51–71)(25/9)	70 (69–71) (22/6)	70(60–81)(22/6)	79 (72–87) (29/7)	72 (63–80) (29/8)
Conditioning regimen	*P* = 0.9	*P* = 0.52			*P* = 0.3	*P* = 0.78	*P* = 0.67	*P* = 0.67	*P* = 0.51	*P* = 0.43
MAC	75(71–79)(141/34)	69(66–70)(141/40)	72 (64–80)(46/10)	71(63–80) (46/11)	81 (74–88)(33/6)	66(57–75)(33/10)	74 (64–83)(24/6)	74(64–83)(24/6)	71 (64–78)(38/12)	65 (58–73)(38/13)
RIC	69 (54–84) (12/3)	75 (60–91) (11/2)	c	c	60 (38–82) (5/2)	75 (54–96) (4/1)	(1/0)a	(1/0)a	83 (81–85) (6/1)	83 (68–98) (6/1)
Donor	*P* = 0.11	*P* = 0.29	*P* = 0.92	*P* = 0.8	*P* = 0.6	*P* = 0.11	*P* = 0.7	*P* = 0.84	*P* = 0.13	*P* = 0.2
MRD	79 (74–84) (73/14)	72(71–73)(73/18)	69 (55–82) (21/5)	69(56–82)(21/5)	69 (55–82) (17/2)	66(54–78)(17/5)	76 (64–88) (13/3)	76(65–88)(13/3)	82 (74–90) (22/4)	77 (68–86) (22/5)
MUD	73 (67–79) (62/16)	72(71–73)(61/17)	75 (64–87) (18/4)	72(62–83)(18/5)	75 (64–87) (18/4)	75(64–86)(17/4)	74 (58–90) (9/2)	74(58–90)(9/2)	65 (53–76) (17/6)	65 (53–76) (17/6)
Haplo	67 (55–78) (18/7)	56(42–69)(18/7)	86 (72–98) (7/1)^b^	86 (72–98) (7/1)	(3/2)^a^	(3/2)^a^	(3/1)^a^	(3/1)^a^	(5/3)^a^	(5/3)^a^

*HLH* Hemophagocytic lymphohistiocytosis, *f-HLH* familial HLH, *STX11* syntaxin 11, *STXBP2* syntaxin binding protein 2, *GS2* Gricelli syndrome type 2, *CHS* Chediak–Higashi syndrome, *OS* overall survival, *EFS* event free survival, *CR* complete remission, *MAC* myeloablative conditioning, *RIC* reduced intensity conditioning, *MRD* matched related donor, *MUD* matched unrelated donor.

^a^Survival analysis could not be performed because the number of patients in the groups was small; only patient numbers and event numbers were given.

^b^The follow-up period was 2 years.

^c^There were no patients receiving RIC.

### Mortality

Of the recipients of the allogeneic grafts (*n* = 37/153 [24%]) who died, 31 died within the first year (25 died within 100 days of post-transplant). Causes of death were infection in 14 (38%) patients, GVHD plus infection in 3 (8%) patients, GVHD in 5 (13.5%) patients, HLH relapse in 7 (19%) patients, pulmonary complications in 4 (11%) patients, VOD in 1 (2,5%) patients and 3 (8%) patients died from other causes. In total, 22 (59,5%) children who died in the first 100 days and 29 (78%) children who died at the end of the follow-up period died from TRM. TRM at day 100 and TRM at the end of follow-up were not statistically significant between genetically different subgroups of patients with HLH (*p* = 0.8 and *p* = 0.36) (Table 5). Multivariate analysis revealed that different mutations had no effect on TRM (*p* = 0.29) (Table 4A).

## Discussion

In this multicenter retrospective study, the effects of genetic mutations on the HSCT process and outcomes were investigated in 153 paediatric patients with HLH who underwent HSCT. There was no statistical difference between the subgroups with genetically different mutations in terms of neutrophil/platelet engraftment, VOD, aGVHD, cGVHD, TRM, OS and EFS rates. Multivariate analyses showed that genetic mutations did not affect engraftment, VOD, aGVHD, cGVHD, TRM, OS and EFS. For children with mutations in PRF1, UNC13D, STX11/STXBP2 and GS2/CHS, 5-year OS post-HSCT values were 72%, 77.6%, 74% and 72.7%, respectively (log-rank > 0.05). Although the survival results of different subgroups of f-HLH were not given in the HLH-1994 study [22], in the HLH-2024 study, 5-year OS values in children with PRF1, UNC13D, STX11 and STXBP2 mutations were 70%, 70%, 80% and 71%, respectively. In the group of patients with GS2/CHS and XLP, 5-year OS and EFS were 70%. OS was significantly lower (52%; *p* = 0.04) and EFS was nonsignificantly lower (52%; *p* = 0.27) for children without verified f-HLH than for children with f-HLH. In this study, pretransplant CR and presence of verified genetic disease were associated with a high survival rate (*p* < 0.05) [22]. The OS and EFS results of our study are similar to those of the HLH-2004 study. In our study, the most important factor affecting OS and EFS was remission status before HSCT.

In the HLH 1994 study, the prognostic impact of age at onset and the interval between the start of treatment and HSCT was analysed, but these factors were not associated with significant alterations in OS. In the HLH-2004 study, the authors reported that prolonged duration of disease activity caused long-term complications [21]. In our study, the age at diagnosis was younger and the time from diagnosis to HSCT was shorter in patients with PRF1 mutations than in the other groups. However, we could not show the effect of these conditions on the HSCT process. In the STX11/STXBP2 group, the age at diagnosis was older and the time to HSCT was longer (*p* > 0.05). However, OS and EFS of those who underwent HSCT at an older age were statistically better.

Although we have achieved satisfactory results in terms of OS and EFS, the TRM rate was high on day 100 and at the end of follow-up in our study, as in many studies [35–40]. In recent years, studies have attempted to develop strategies to reduce toxicity. Several studies have examined the effectiveness and toxicity of drugs such as emapalumab [41], ruxolitinib [42, 43] or alemtuzumab [44], instead of etoposide, the most toxic drug of HLH-2004, to achieve remission until HSCT. In recent years, efforts have been made to reduce the toxicity of the conditioning regimen in HSCT by administering RIC/RTC instead of the MAC regimens that have been recommended as a standard for many years [24–26, 30]. In our cohort, 133/153 patients received the HLH 2004 protocol until HSCT, and 92% of the patients received the MAC regimen as conditioning. It may be associated with high TRM. Because of the use of the MAC regimen in our cohort, engraftment rate and donor chimerism rate were high, and the rates of primary/secondary graft failure, HLH relapse, and need for secondary cellular treatments were not as high as those for the RIC/RTC regimens [45, 46]. Although VOD is associated with high TRM in many studies [35–38], the mortality rate due to VOD was lower (2.5%) in our study. This may be associated with high rates of prophylactic defibrotide use. However, we could not demonstrate statistical superiority of prophylactic defibrotide use in preventing VOD or reducing its severity.

There are some limitations of this study. The study is retrospective, genetic testing could not be performed on some patients, and not all mutations were examined in some patients. 38 patients for whom we had HSCT data were excluded from the study. If more comprehensive genetic analyzes such as targeted next-generation sequencing (NGS), whole exome sequencing (WES), or whole genome sequencing could be performed as currently recommended [47], novel mutations known to cause HLH could be detected in some patients. In this way, our patient number or genetic subgroup number could have been higher. However, even with these techniques, molecular diagnosis may remain unsolved in some individuals with presumed p-HLH. In a recent study, a targeted NGS panel approach containing 15 HLH-related genes was used in 1892 patients with suspected p-HLH, and a definite genetic diagnosis could be made in only 10.4% of the patients (*n* = 197). Potential causal genetic findings were found in 12% of the patients (*n* = 227) in this study [48]. By contrast, Chinn et al. identified HLH-associated genes in 58% of cases by WES in patients selected by HLH-2004 criteria and flow cytometry-based immunological screening [47].

In conclusion, in our study, similar to the results in the literature, the most important factor affecting OS and EFS was remission status before HSCT. Although different rates were detected between genetic subgroups in terms of HSCT outcome, the results were not statistically significant. The most serious problem was high TRM, especially in the first 100 days. The most common causes of death was infection and GVHD. Therefore, the success of HSCT in children with HLH may be increased by developing new treatment protocols that achieve remission without increasing toxicity and by treating infections more effectively. However, in future prospective studies that include more patients and create new genetic subgroups by performing more detailed genetic analyses, specific recommendations for genetic subgroups of HLH can be defined.

## Data Availability

The datasets generated during and/or analysed during the current study are not publicly available due [The data belong to the centres of the Turkish Paediatric Bone Marrow Transplantation-Study Group] but are available from the corresponding author on reasonable request.
